# Minding the gap between theory and clinical practice: an individualised approach to the management of feline diabetes mellitus

**DOI:** 10.1186/1751-0147-57-S1-P6

**Published:** 2015-09-25

**Authors:** Moira S Lewitt, Emma Strage, David Church

**Affiliations:** 1 School of Health, Nursing and Midwifery, University of the West of Scotland, Paisley, UK; 2 Department of Clinical Sciences, Swedish University of Agricultural Sciences, Uppsala, Sweden; 3 Department of Veterinary Clinical Sciences, Royal Veterinary College, University of London, London, UK

## Introduction

Insulin independence is a realistic therapeutic goal in feline diabetes. Remission is dependent on achieving fast glycemic control. However, without intensive monitoring high insulin doses increase the risk of hypoglycemia. An 11-year old spayed Burmese cat presented with diabetes after steroid treatment for skin allergy. Control of blood glucose was not achieved using low carbohydrate diet plus the recommended q12h treatment with either insulin lente or glargine, with the total dose limited by the risk of hypoglycaemia.

## Objectives

The aim was to achieve euglycaemia and insulin-independence.

## Methods

A more frequent insulin treatment was tried. Seven weeks from start of q12h treatment, the total daily dose of insulin glargine was divided across three injections. One week later the frequency of injection was increased to q6h. There was intensive monitoring of capillary blood glucose (Accu-Chek Aviva).

## Results

There was a prominent glucose nadir after approximately 4h regardless of insulin type. Within four weeks on insulin q8h and q6h blood glucose levels were within the normal range. Weight gain was noted and within one week of caloric restriction insulin was withdrawn completely. One month later fructosamine had normalized and after more than three years the cat remains off insulin with blood glucose levels at the upper limit of the reference range.

## Conclusions

More frequent insulin injections than recommended in literature may be necessary to achieve glycemic control. Owners are important collaborators in feline diabetes care and with intensive home monitoring more frequent insulin treatment may lead to remission without hypoglycemia.

